# Observations on the Cure and Prevention of the Contagious Fever Now Prevalent in the City of Edinburgh and Its Environs, &c. &c.

**Published:** 1818-06

**Authors:** 


*Observations on the Cure and Prevention of the contagious Fever now prevalent in the City of Edinburgh and its Environs, &c. &c.* By J. Yule, M. D. F. R. S. Edin. Edinburgh, 1818. pp. 58.

This pamphlet, as the title expresses, is confined almost entirely to prophylaxis, including etiology and treatment. In respect to the origin of the fever, Dr. Yule advocates its domestic source, especially from an atmosphere contaminated by the breath and other exhalations of a too crowded population. After relating some cases, and tracing their history, Dr. Y. observes,

"In these instances, then, we have facts which strongly demand attention. Typhous poison, originating evidently from the bodies of a number of persons crowded in a damp, close apartment in a sunk area, speedily ascending to the family immediately above;—soon after affecting the family in the garret; and in the course of ten weeks, the twelve individuals under the same roof." P. 13.

In other parts of the city, Dr. Yule remarks, that similar instances might be added, illustrating clearly "the domestic origin of the contagion." *ib.*

Dr. Yule appears to think, that there is no doubt respecting the introduction of *yellow fever contagion* into North America from the West Indies! He also appears quite certain that the fumes of acids will *arrest the progress* of contagion! He is more judicious, when he speaks of the means of preventing the generation of the contagion, though he brings forward nothing that has not been repeatedly stated by many others.

*Treatment.* The free admission of pure air; the application of cold to the fevered surface; blood-letting, mercury, &c. are cursorily noticed. Blood-letting, he thinks, is a practice in this fever which, with few exceptions, seems of doubtful utility. Dr. Yule's opinion on this point seems to rest on that of his friend Dr. Wright.

"His great experience, no doubt, occurred between the tropics, where the climate powerfully accelerates the fate of the sick, and *the rapid career of the disease does not admit of bleeding.*" P. 42.

We are not a little surprised to find an Edinburgh Physician so totally unacquainted with the medical literature of modern times, as to issue forth a sentence like the above, which almost every Tyro in the profession could prove to be erroneous. Between the tropics, it is *unani-*

*mously* agreed, that early and liberal venæsection is the *sine qua non* of cure; and therefore, when we see a prohibition of venæsection in the fevers of this country, grounded on a *false* conclusion respecting the fevers of another, it impresses us with no very favourable ideas of either the logical or practical deductions of the author. We would recommend Dr. Yule to dip into our highly-respected contemporary, *The Edinburgh Journal,* for information relative to the depletory treatment in West India fevers; and into the writings of Drs. Armstrong and Dickson, for the success which attended venæsection in the typhous fevers of this country.

"But there is happily another remedy which, in my experience at least, can be used with invariable advantage, when bleeding would be attended with the utmost hazard—MERCURY. The free use of this invaluable remedy, in the very worst forms of acute diseases, would seem, like many of the most useful of the sciences, to have originated in the East. On his [Dr. Wright's] authority, I first began the use of it in the worst forms of contagious fever, occasionally occurring in this city; and, when early administered, with constant success, especially when combined with the necessary action of cold, the extent, as well as the medium of which, being, of course, regulated by the circumstances of individual cases." P. 44.

Dr. Yule's observations on the other therapeutical items contain nothing new or detailed. The whole pamphlet seems more designed for the general reader than for the physician; and therefore consists of prophylactic and etiological discussions, rather than pathological and therapeutic. Upon the whole, we were a good deal disappointed in our hopes of professional information on a subject of considerable import, both in this and the sister kingdom.

